# Proteomic study of *Desulfovibrio ferrophilus* IS5 reveals overexpressed extracellular multi-heme cytochrome associated with severe microbiologically influenced corrosion

**DOI:** 10.1038/s41598-021-95060-0

**Published:** 2021-07-29

**Authors:** Mohor Chatterjee, Yu Fan, Fang Cao, Aaron A. Jones, Giovanni Pilloni, Xiaozhou Zhang

**Affiliations:** 1 Corporate Strategic Research, ExxonMobil Research and Engineering Company, 1545 Route 22 East, Annandale, NJ 08801 USA; 2 ExxonMobil Technical Computing Company, 1545 Route 22 East, Annandale, NJ 08801 USA

**Keywords:** Mass spectrometry, Microbiology techniques, Proteomic analysis, Proteins, Proteomics, Applied microbiology, Environmental microbiology, Industrial microbiology

## Abstract

Microbiologically influenced corrosion (MIC) is recognized as a considerable threat to carbon steel asset integrity in the oil and gas industry. There is an immediate need for reliable and broadly applicable methods for detection and monitoring of MIC. Proteins associated with microbial metabolisms involved in MIC could serve as useful biomarkers for MIC diagnosis and monitoring. A proteomic study was conducted using a lithotrophically-grown bacterium *Desulfovibrio ferrophilus* strain IS5, which is known to cause severe MIC in seawater environments. Unique proteins, which are differentially and uniquely expressed during severe microbial corrosion by strain IS5, were identified. This includes the detection of a multi-heme cytochrome protein possibly involved in extracellular electron transfer in the presence of the carbon steel. Thus, we conclude that this newly identified protein associated closely with severe MIC could be used to generate easy-to-implement immunoassays for reliable detection of microbiological corrosion in the field.

## Introduction

Microbiologically influenced corrosion (MIC) is recognized as a considerable threat to asset integrity in the oil and gas industry. Approximately 20% of the total cost of corrosion in oil field operations is estimated to be associated with MIC^1^. Although vast amounts of academic knowledge and industrial experience exist, an effective and economic approach to diagnose and monitor MIC is still lacking^1,2^. Current MIC diagnosis and monitoring techniques (e.g. counting the total number of living cells in a given sample, the detection of sulfate-reducing bacteria (SRB), and microbial community analysis) are either expensive, slow, or highly uncertain. False conclusions may result from the use of these techniques since the presence of broad groups of bacteria does not mean that corrosion-inducing metabolisms are occurring at the site of sampling. Furthermore, the correlation between these parameters and corrosion is still poorly understood, and may even be system-specific or in some cases non-existent. There is an urgent need for a more reliable and broadly applicable method for MIC monitoring and diagnosis in the field that can enable informed decision-making on MIC mitigation as well as confirm the effectiveness of an implemented MIC mitigation program in the field.

**Figure 1:**
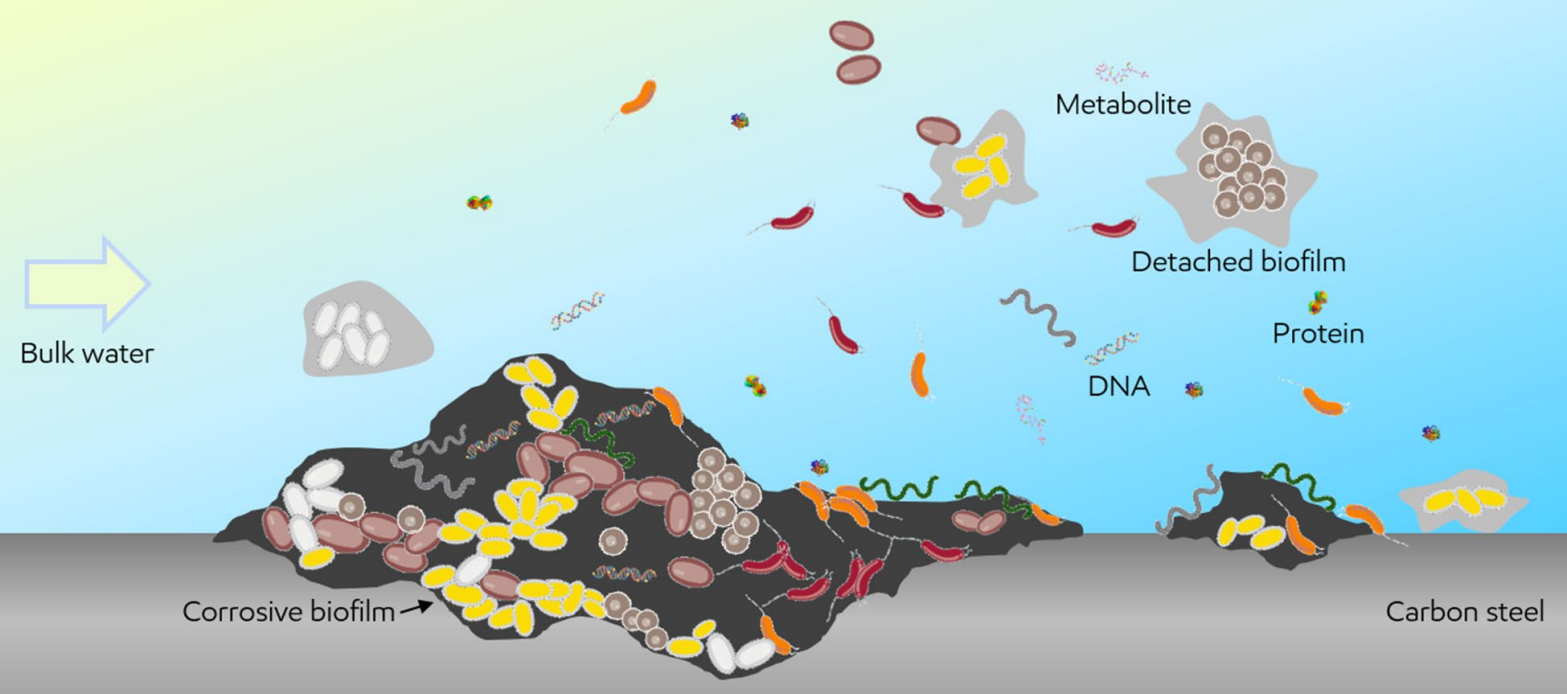
Schematic illustration of corrosive biofilm and potential biomarkers (e.g., protein, DNA, and metabolite) associated with MIC.

MIC occurs when microorganisms attach to the inner wall of pipelines or other ferrous oil field infrastructure to form biofilms (Fig. 1) and locally consume the metal either directly or as a collateral effect of corrosive metabolic by-products (e.g., hydrogen sulfide). Many microorganisms involved in MIC can additionally take up electrons from steel through membrane-associated redox proteins via extracellular electron transfer mediators (such as proteins, matrix of extracellular polymeric substances (EPS), and corrosion products)^3,4^ (Fig. 1). Furthermore, certain types of microbial metabolism can lead to corrosion of the metallic substrate on which the biofilm develops^5^. The rationale of using proteomic profile from either intracellular or extracellular sources for MIC diagnosis and monitoring lies in the fact that corrosive microorganisms undergo unique metabolisms and produce signatures in the surrounding environment which would not occur with non-corrosive microorganisms. By establishing the relationship between the type, relative abundance, and/or pattern of proteins with the type and severity of MIC, a culture-independent and reliable MIC diagnosis and monitoring approach can be developed.

MIC in seawater environments is a serious environmental hazard. It has been reported previously that SRB are responsible for localized corrosion of metal infrastructures in a nutrient deficient environment like seawater^6,7^. In seawater, microorganisms can directly use metallic iron as an electron donor and aggressively attack the metal infrastructure. This is called electrical MIC or EMIC. *Desulfovibrio ferrophilus* strain IS5 is reported to be a lithotrophic organism isolated from a seawater environment that is capable of an EMIC mechanism^5,7^. EMIC processes also cause higher corrosion rates of steel (up to 0.7 mm/y) compared to other MIC mechanisms^3,4,8^. Therefore, it is of significant interest to study the mechanisms of EMIC occurring with *D. ferrophilus* strain IS5 grown under nutrient limited conditions. This work is the very first study to focus on MIC biomarker discovery with *D. ferrophilus* strain IS5 by using untargeted proteomics.

## Results and discussion

### Microbiologically influenced corrosion by *Desulfovibrio ferrophilus* IS5

Bacterial reduction of oxidized sulfur species is critical for energy production in anaerobic marine and subsurface environments. It was identified that extracellular outer-membrane cytochromes are broadly conserved in sediments containing sulfur-respiring bacteria and enable cells to directly use electrons from insoluble minerals via extracellular electron transport^3,4,9^. *Desulfovibrio ferrophilus* strain IS5 is one such strain that can cause severe corrosion of iron through EMIC^7^.

**Figure 2:**
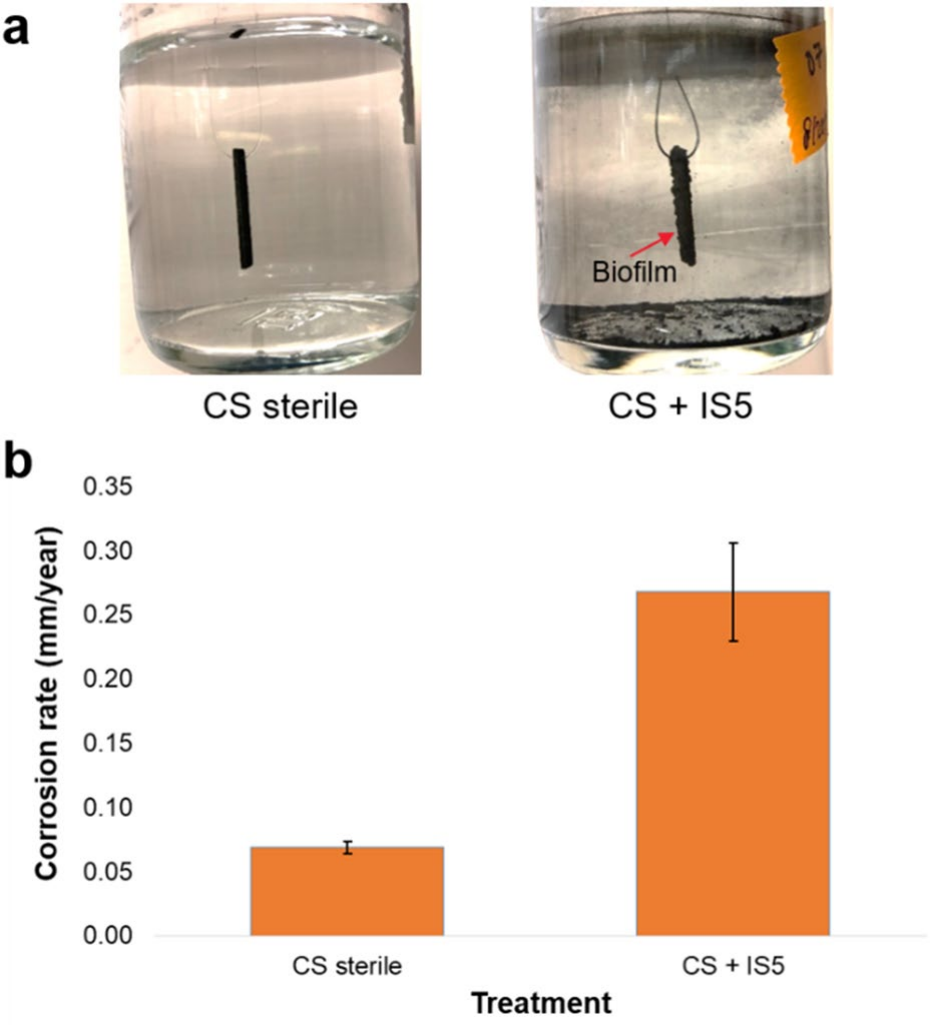
Visualization of the carbon steel coupons and their corrosion rate. (**a**) Visualization of the carbon steel coupons after 27 days of incubation without (CS sterile) and with (CS + IS5) *D. ferrophilus* IS5; (**b**) corrosion rates of the carbon steel coupons under different conditions. The error bars represent the calculated standard deviations of the measurements of three biological replicates.

Obtaining extracellular proteomic profiles will aid in the development of a quick MIC diagnosis technology. For example, the insights gained from such a study could be used to implement immunoassays. In the future, collection of field water samples may allow for the detection of MIC-related proteins to differentiate microbial function specific signatures instead of just detecting the number or types of microorganisms present. With this in mind, to study proteomic profiles in the extracellular environment, *D. ferrophilus* IS5 was cultivated anaerobically with 10 mM formate and 1 mM acetate as organic carbon source and initial energy source, and 27 mM sulfate as the electron acceptor, with either carbon steel (CS) or polyether ether ketone (PEEK) coupons. CS coupons provided surface for electric MIC and PEEK coupons were selected as a control surface where ideally no active electric MIC should take place. The metabolic profiles for formate consumption and sulfate reduction are shown in Fig. S1. After 27 days of incubation, as seen in Fig. 2a, the surface of the carbon steel coupon in the sterile control was still smooth (CS sterile). The surface of the carbon steel coupon which was inoculated with strain *D. ferrophilus* IS5 showed a rough surface with build-up of biofilm and corrosion products (Fig. 2a, CS + IS5). The measured corrosion rate for the carbon steel coupon without and with the existence of *D. ferrophilus* IS5 were 0.07 mm/y and 0.27 mm/y, respectively (Fig. 2b). This measured corrosion rate in the presence of *D. ferrophilus* IS5 is considered to be severe MIC under lab bottle test conditions^10^.

### Characterization of the CS coupon

**Figure 3:**
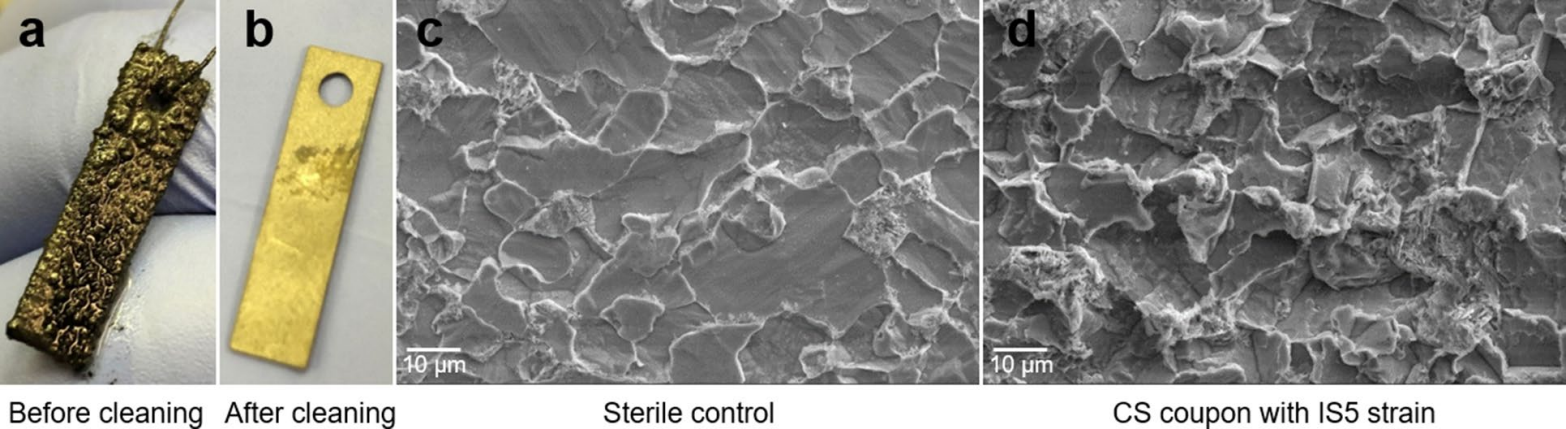
Surface characterization of the corroded coupon. (**a**), the close-up view of the corroded coupon covered with biofilm and corrosion products; (**b**), the close-up view of the surface of the cleaned coupon; (**c**) and (**d**), scanning electron microscope image of the cleaned coupon surface without and with IS5 strain, respectively.

A close-up view of the carbon steel coupon incubated with *D. ferrophilus* IS5 showed a rough and bumpy surface due to the buildup of biofilm and corrosion products (e.g. pyrrhotite and siderite) on the surface (Fig. 3a). After the removal of the biofilm and corrosion products, no obvious pitting was observed on the surface of the corroded coupon (Fig. 3b). This observation indicated that corrosion caused by *D. ferrophilus* IS5 in this study was of a general, uniform type rather than of a localized type. The surface of the coupon was examined with scanning electron microscope (SEM), as shown in Fig. 3c,d. The coupon in the sterile control without IS5 strain showed mild general corrosion with a fairly flat surface as shown in Fig. 3c. In contrast, a large undulated surface was observed on the steel coupon with IS5 strain (Fig. 3d) due to uneven corrosion of individual grains with different crystallographic orientation, suggesting a higher general corrosion rate than the sterile control without IS5. This is consistent with the corrosion rate measurement result as shown in Fig. 2.

### Characterization of the biofilm with scanning electron microscope

**Figure 4:**
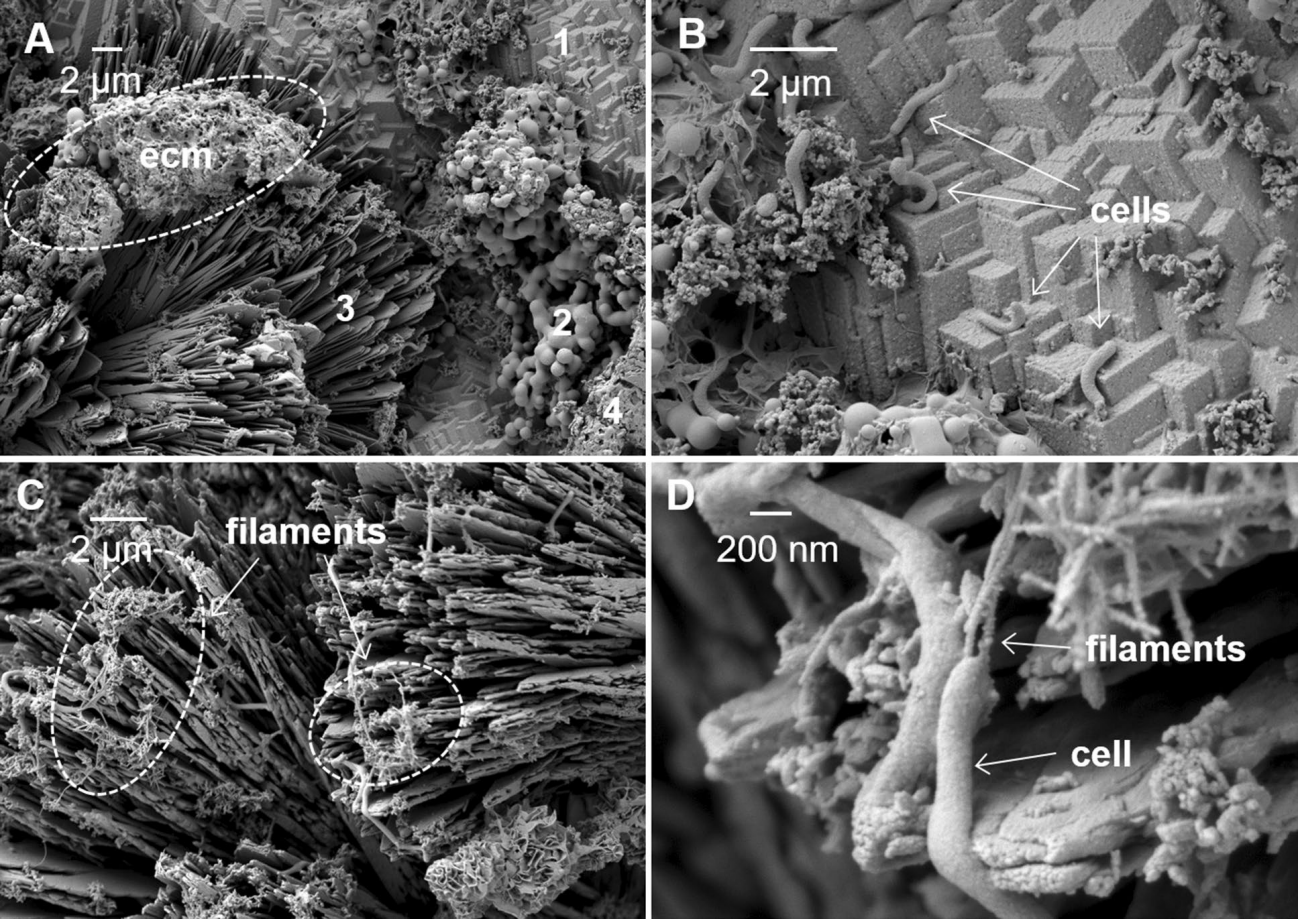
Scanning electron microscopy (SEM) micrograph images of *D. ferrophilus* IS5 biofilms attached to the surface of and actively corroding a carbon steel coupon. Scale bars for images (**A**), (**B**), and (**C**) are all 2 µm and scale for (**D**) is 200 nm. Panel (**A**) shows individual cells and biofilms with associated extracellular matrix **(ecm, dashed oval)** attached to a morphologically diverse assortment of at least four different types of mineral crystals **(1, 2, 3, and 4)** formed during microbial corrosion. Panel (**B**) is an enhanced view of the upper right quadrant of panel (**A**) identifying individual cells of *D. ferrophilus IS5* attached to the surface. Panel (**C**) shows cells attached to mineral crystals with filamentous structures forming connecting bridges between individual cells and crystals. Panel (**D**) is an enhanced view of filamentous protrusions associated with and extending from individual cells forming bridges between cells and the corroded surface.

SEM imaging of the biofilm showed cells of *D. ferrophilus* IS5 attached to the corrosion products and minerals of at least four different morphologies (Fig. 4). Furthermore, extracellular nano-filamentous structures with diameters of about 50 nm were observed to extend between cells and the surface of the corrosion products (Fig. 4C,D). This is a similar observation to a previous report in which strain IS5 had cytochromes detected on the surface of nanowires with a similar diameter^9^.

### Corrosion product analysis

X-ray diffraction (XRD) analysis of the residual corrosion products on steel coupon surface after biofilm removal suggests the presence of pyrrhotite (Fe_7_S_8_) and siderite crystalline phases on the steel coupon surface, as shown in Fig. S2. Similar phases were detected by XRD on the filtered out corrosion product sample from the acetone phase after sonicating. Scanning electron microscopy (SEM) analysis of the coupon surface revealed two morphologically-distinct corrosion products with different crystal sizes, as shown in Fig. S3. Energy dispersive X-ray spectroscopy (EDS) elemental mapping, as shown in Fig. S4, indicates that the small powder-like crystals with white contrast in the backscattered electron (BSE) image are rich in Fe and S, while the globular crystals with dark contrast are rich in O, Ca and Mg to some extent. Combining SEM/EDS and XRD results together, the small powder-like crystals are identified as pyrrhotite and the globular crystals are identified as a mixture of FeCO_3_ and Ca_0.1_Mg_0.33_Fe_0.57_CO_3_. Siderite was also identified in the corrosion products of iron coupons exposed to anoxic environments with IS4 strain by Enning et al*.*^7^. However, amorphous ferrous sulfide rather than pyrrhotite was reported to be the sulfur containing corrosion products with the IS4 strain.

### Proteomics analysis

**Figure 5:**
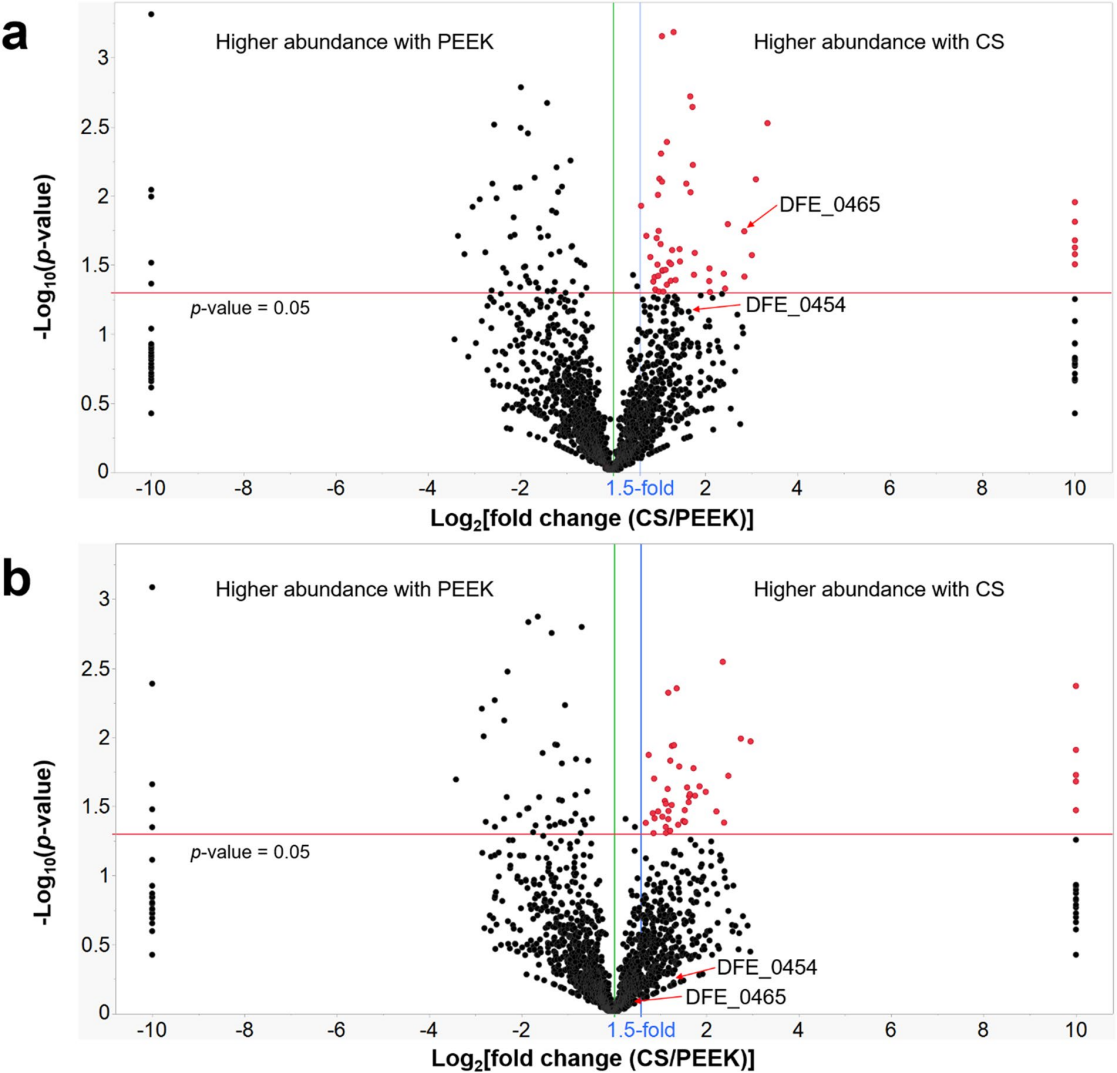
Volcano plots representing the results of the proteome analysis of proteins in the extracellular environment (**a**) and planktonic cell proteins (**b**) with *D. ferrophilus* IS5 cultured with either CS or PEEK coupons. Highlighted (red) points represent proteins showed higher abundance when the culture was incubated with the CS coupon and showed severe EMIC, with statistical significance. Red lines represent the applied significance thresholds of a t-test *p*-value = 0.05. Blue lines represent the applied thresholds of an absolute fold change ≥ 1.5. Details are shown in Table 1 and the Supplementary Information.

Proteomic profiles of the total extracellular proteins of IS5 strain, which was cultivated for 27 days, were analyzed by liquid chromatography with tandem mass spectrometry (LC–MS/MS). In total, 2192 proteins were identified. Fold change of proteins (CS vs. PEEK) versus the statistical significance, as *p*-value, of the data was plotted as a volcano plot (Fig. 5). For the extracellular proteomic profiles, as showed in Fig. 5a, 57 proteins showed significant overexpression (which were highlighted in red, fold change > 1.5-fold, *p*-value < 0.05) when the culture was incubated with CS coupon, and 7 out of these 57 proteins were unique to CS incubation. It was recently shown that *D. ferrophilus* IS5 performs extracellular electron uptake through direct cell contact via the biofilm on the cathode surface^11^. In this study, many overexpressed proteins in CS treatment are involved in cellular processes such as electron transfer, protein transport, protein complex formation, chemotaxis, biofilm development, and stress response (Table 1). The observed overexpressed proteins may indicate active biofilm development and extracellular electron transfer when cells are exposed to CS under organic electron donor starvation conditions^8^. The results may also indicate an active EMIC process through chemotaxis, biofilm development, and extracellular electron transfer in the CS treatment under organic electron donor-starved conditions, which is consistent with the observation of a thick biofilm on the CS coupon (Fig. 2a) in contrast with no obvious biofilm formation on the PEEK coupon. It was noticed that many identified proteins from the extracellular environment are typical cytosol proteins. These cytosol proteins may result from the cell lysis with the long cultivation period of this study. It is also possible that some cytosol proteins may get to the extracellular environment through unknown non-classical secretion pathways^12,13^.

**Table 1:** Selected significantly overexpressed proteins by *D. ferrophilus* IS5 which may be correlated to MIC activity.

Protein ID	Protein description	Fold change^a^ (CS/PEEK)	*p*-value^b^	Function	Secreted? Secretion Type?
DFE_2481	Chemotaxis protein methyltransferase	INF	0.015	Methylation of the membrane-bound methyl-accepting chemotaxis proteins	
DFE_1378	MotA/TolQ/ExbB proton channel	INF	0.026	Protein transport	
DFE_2306	Putative endoribonuclease	INF	0.015	Putative endoribonuclease	
DFE_2414	Peptide ABC transporter ATPase	INF	0.021	Peptide transport	
DFE_0002	Putative Transposase	INF	0.024	Putative transposase	
DFE_3359	UspA domain protein	INF	0.031	Universal stress protein	
DFE_1992	Uncharacterized protein	10	0.003	Transmembrane protein, potential pili subunit	
DFE_0727	Uncharacterized protein	7.2	0.038	Unknown	Yes, Signal peptide (Sec/SPI)
DFE_0465	Multiheme cytochrome	7.2	0.018	Electron transfer	Yes, Signal peptide (Sec/SPI)
DFE_3065	Tetratricopeptide repeat repeats containing protein	4.3	0.05	Forms structural scaffolds to mediate protein–protein interactions and often the assembly of multiprotein complexes	
DFE_1264	Hydrogenase maturation factor	3.3	0.006	Cellular processing	
DFE_0334	Putative LpxI-like phosphosaccharolipid hydrolase	2.2	0.044	Hydrolysis of UDP-2,3-diacyl- glucosamine for the biosynthesis of lipid A	
DFE_2738	Uncharacterized protein	2.1	0.049	Unknown	Yes, Lipoprotein signal peptide (Sec/SPII)
DFE_3147	Oxidoreductase, FAD/4Fe-4S ferredoxin-binding protein	2.1	0.034	Electron transfer	
DFE_2290	Uncharacterized protein	2.1	0.035	Unknown	
DFE_3364	4Fe-4S ferredoxin	1.6	**0.069**	Electron transfer	Yes, TAT signal peptide
DFE_3267	Putative redox-active protein (C_GCAxxG_C_C family, putative FeS cluster-binding)	2.1	**0.07**	Electron transfer	
DFE_1193	Uncharacterized protein	INF	**0.056**	Unknown	

^a^INF means fold change is infinity since the protein is only detected with CS.

^b^Identified proteins with *p*-value > 0.05 were highlighted in bold.

**Figure 6:**
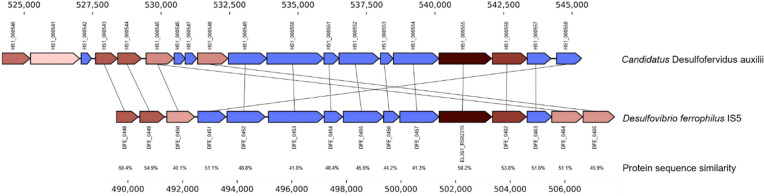
The cytochrome cluster and the associated proteins from the genome of *D. ferrophilus* IS5. Genes are colored the same between organisms, with the exception of the cytochromes, which are colored with various intensities of red based on the number of heme binding motifs present in the gene.

Deng et al*.* recently identified a cluster of multi-heme cytochromes from the sequenced genome of strain *D. ferrophilus* IS5^9^. It is noticed that this cytochrome cluster is absent in other *Desulfovibrio* that corrode primarily by a chemical MIC mechanism (e.g. *Desulfovibrio alaskensis* G20, *Desulfovibrio vulgaris* Hildenborough)^5^. The genes within this cluster resemble the pattern for reportedly large multi-heme cytochrome cluster from many different microorganisms which are capable of performing extracellular electron transfer between the cells or the direct electron uptake from Fe^(0)^^14^. The detailed structure of the cluster of multi-heme cytochromes from IS5 is shown in Fig. 6 with the prediction for function and existence of secretion signal peptides shown in Table 2. Similar cluster of multi-heme cytochromes from *Candidatus* Desulfofervidus auxilii, which was proposed to be involved in direct extracellular electron transfer^14^ are shown in Fig. 6. Homologs of four of the genes in this cluster, predicted to form a peptidyl-propyl-cis–trans isomerase (DFE_0457), two multi-heme *c*-type cytochromes (EL361_RS02315 and DFE_0462) and a beta propeller fold protein (DFE_0463), respectively, have been recently shown to be conserved in cytochrome gene clusters from other organisms that are known to perform direct electron transfer reactions^14^. Different extracellular electron transfer processes by the organisms shown in Fig. 6 includes direct interspecies electron transfer or mineral respiration, which are reflected by the different types of clusters found in those organisms. There are, in total, 15 proteins shown in the cluster and 7 of them were predicted as heme-containing proteins. Fourteen of these 15 proteins were predicted to have the secretion signal peptide and 6 of them are secreted membrane-bound lipoproteins. It is shown in the volcano plot (Fig. 5) and Table 2 that most of the proteins in the multi-heme cluster were detected with the proteomics analysis and one of these multi-heme cytochromes (protein ID DFE_0465) is significantly overexpressed under the electron donor limited conditions with the CS coupon. This observation was consistent with the observation by Deng et al*.* which was a result of a transcriptomics study^9^. Deng et al*.* also detected the overexpression of other multi-heme cytochromes (protein ID: DFE_0449, DFE_0450, DFE_0461, and DFE_0464) through transcriptomics analysis and protein sequence analysis with outer-membrane cytochromes in the crude membrane fractions. In this proteomics study, we also detected the expression of DFE_0450, DFE_0461, and DFE_0464 (Table 2). However, these specific cytochromes were not determined to be significantly overexpressed under electron donor limited conditions in our proteomics study. Compared with the proteomics study by McCully et al. on an electron-conductive biofilm, we detected the [Fe]- and [NiFe]-hydrogenases subunits (DFE_2058 and DFE_3279) from the biofilm and planktonic proteomic profiles, and they are not overexpressed under corrosive conditions. The two remaining proteins that could have been associated with direct electron uptake (DFE_1038 and DFE_2965), were not detected in our proteomic study. The above observation of differential proteomic expression in our study from McCully et al.’s study may be due to different experimental conditions.

**Table 2:** Description of the proteins and their secretion signal peptide prediction for the putative cytochrome island containing large multi-heme cytochromes from *D. ferrophilus* IS5.

Protein ID	Protein description	Signal peptide?	Detected with planktonic cells?	Detected with biofilm cells?	Detected with extracellular proteins?
DFE_0448	Multiheme cytochrome *c*	Sec			
DFE_0449	Multiheme cytochrome *c*	Sec, lipoprotein SP			
DFE_0450	Multiheme cytochrome *c*	Sec	Y		Y
DFE_0451	6-Bladed beta-propeller structure of unknown function	Sec	Y		Y
DFE_0452	7-Bladed beta-propeller fold protein of unknown function	Sec, lipoprotein SP	Y	Y	Y
DFE_0453	F5/8 type C domain protein		Y	Y	Y
DFE_0454	ABC-type transport auxiliary lipoprotein component	Sec, lipoprotein SP	Y		Y
DFE_0455	OmpA family protein	Sec	Y		Y
DFE_0456	Lipoprotein	Sec, lipoprotein SP	Y		
DFE_0457	Peptidylprolyl cis–trans isomerase	Sec	Y	Y	Y
EL361_RS02315	Cytochrome *c* nitrite reductase pentaheme subunit, part of an anaerobic dimethyl sulfoxide reductase	Sec, lipoprotein SP	Y	Y	
DFE_0462	Multiheme cytochrome *c*	Sec			Y
DFE_0463	Six-bladed beta-propeller fold protein	Sec, lipoprotein SP	Y		Y
DFE_0464	Multiheme cytochrome *c*	Sec		Y	Y
DFE_0465	Multiheme cytochrome *c*	Sec	Y		Y

The signal peptides were predicted by SignalP-5.0 Server (http://www.cbs.dtu.dk/services/SignalP/).

To understand the spatial distribution of the cytochrome DFE_0465 within a cell, proteomic profiles of biofilm cells, planktonic cells, and total proteins in the extracellular environment were also analyzed (Fig. 5). Interestingly, cytochrome DFE_0465 was only statistically significantly overexpressed (7.2-fold, *p*-value = 0.018) within protein profile for the extracellular environment (Fig. 5a). For the proteomics profile of the biofilm cells, cytochrome DFE_0465 was not detected (Fig. S5 and Supplementary Information). Based on the above results, it is reasonable to anticipate that the cytochrome DFE_0465 was secreted outside of the cell (instead of staying within the periplasmic space) as an extracellular mediator for electron transfer. This cytochrome might be loosely associated with the cell and was rinsed away when we collected the cells from the CS coupon through sonication. As shown in Fig. 5, the membrane-bound lipoprotein which was predicted to have a secretion signal peptide (DFE_0454, a potential ABC-type transport auxiliary lipoprotein component) was also overexpressed in proteomic profiles for both the planktonic cells and the extracellular proteins but not at a statistically significant level.

## Conclusions

Many microorganisms involved in MIC can take up electrons from steel through membrane-associated redox proteins via extracellular electron transfer mediators (such as proteins, matrix of extracellular polymeric substances (EPS), and corrosion products)^3,4,15,16^. These extracellular electron transfer mediators may serve as good biomarkers for more convenient MIC diagnosis and monitoring in oil fields^17^. It is of significant interest to study the mechanisms of EMIC since it is believed to be one of the major culprits for the steel pipeline degradation that is seen in oil fields. In this study, *D. ferrophilus* IS5 strain was cultivated under nutrient limited conditions to induce severe EMIC. The comprehensive proteomic profiles (planktonic cells, biofilm cells, and proteins in the extracellular environment) were characterized for this EMIC-causing strain. Many overexpressed proteins in corrosive conditions which are involved in cellular processes such as electron transfer, protein transport and complex formation, biofilm development, and stress response are identified. Interestingly, the cytochrome DFE_0465 from the conserved large multi-heme cytochrome cluster was found to be significantly overexpressed in the extracellular proteomic profile under the severe MIC conditions, which could be a promising MIC biomarker candidate. Further verifications, ideally in a simulated field condition, will shed more light on the possibility of DFE_0465 as a possible MIC biomarker. It is anticipated that once the protein-based MIC biomarker is verified, the corresponding immunoassay can be further developed for quick, convenient, and reliable MIC diagnosis and monitoring in the oil field, which is based on what the microorganisms in the field are doing instead of the type of microbes present.

## Materials and methods

### Growth of *Desulfovibrio ferrophilus* strain IS5

Artificial seawater medium was used to culture *Desulfovibrio ferrophilus* strain IS5 and was prepared by adding the following components (all from Sigma-Aldrich, St. Louis, MO, USA) to 1 L MilliQ water: CaCl_2_ (1.5 g), NaCl (26.25 g), KCl (0.6 g), MgCl_2_ (5.7 g), and MgSO_4_ (6.75 g). pH of the medium was adjusted to 7.2. The liquid was purged with CO_2_:N_2_ (15:85, v/v) for 40 min, and the bottles were equipped with butyl stoppers and O-ring screw caps, through which ~ 180 ml of headspace gas was removed prior to autoclaving at 121 °C for 40 min. After autoclaving and cooling the medium, 1 mL of the following stock solutions were added anaerobically^18^: 0.02% resazurin solution, trace metals, vitamin mixture, thiamine, riboflavin, vitamin B_12_, selenite-tungstate solution, 1 mM phosphate solution and 2 mM ammonium chloride solution. 0.5 mM final concentration of Na_2_S solution was added as reducing agent and 1 mM final concentration of sodium acetate was added as carbon source anaerobically as well. A sterile sodium formate solution was added anaerobically if needed, from a separate stock, to a final concentration of 10 mM. 80 mL of this medium was dispensed into sterile 120 mL serum bottles equipped with butyl stoppers and crimped with aluminum rings. These bottles had 600 grit polished X-52 carbon steel (CS) coupons or polyether ether ketone (PEEK) coupons hung suspended from the neck of the serum bottle via a thin nylon wire piercing the butyl stopper of the bottle. Each cleaned coupon (5 × 20 mm) was weighed in triplicate and average weight was recorded prior to serum bottle assembly. Assembled bottles and holders were flushed with 85%:15% N_2_:CO_2_ through a heated, H_2_ reduced, copper furnace (Supelco) for 20 min and then autoclaved prior to sterile medium addition. *D. ferrophilus* strain IS5 (DSM No. 15579) was obtained as a freezer stock from DSMZ and revived anaerobically with DSMZ medium 195c per DSMZ instructions. Anaerobic freezer stocks were made from this culture with 10% glycerol. 1 mL of a freezer stock was transferred into 40 mL artificial seawater media as detailed above containing a CS coupon, 10 mM formate (electron donor), 1 mM acetate (carbon source) and 27 mM sulfate (electron acceptor). After active sulfate reduction was observed, a 5% inoculum from this culture was transferred into fresh artificial seawater media without formate but with a CS coupon, 1 mM acetate and 27 mM sulfate. The culture was transferred twice under the same conditions every 3 weeks to dilute any residual formate or glycerol that may act as carbon source and sulfate depletion was monitored by anion exchange chromatography. Subsequently, this culture was used as inoculum for determination of microbial corrosion by *D. ferrophilus* strain IS5. The final experiment consisted of strain IS5 artificial seawater medium with 10 mM formate and 27 mM sulfate as electron donor and acceptor along with 1 mM acetate and a CS coupon in a 120 mL media bottle with 80 mL media. Biological triplicates were set up as follows: CS-sterile, CS-inoculated with *D. ferrophilus* strain IS5 and PEEK-inoculated with *D. ferrophilus* strain IS5. Bottles were incubated at 30 °C and 60 rpm for 27 days, sulfate utilization was monitored on days 7 and 27 by anion exchange chromatography. On day 27, proteins from extracellular filtrate, planktonic cells, and biofilm cell samples were collected for proteomics analysis by the Proteomics & Mass Spectrometry Core at Princeton University.

### Time dependent anion monitoring by ion chromatography

Duplicate anion quantification using high pressure ion chromatography (HP-IC) was applied on 1 mL of subsamples collected as needed. A HP-IC 5000 Reagent Free system (Thermo-Fisher Scientific, Massachusetts, USA) equipped with Dionex IonPac AS 11-HC column, EluGen III Potassium Hydroxide capillary cartridge and suppressed conductivity detector Dionex ACES 300, was used for the analyses. Samples were filtered through 0.2 μM cellulose acetate filters and centrifuged at 5000 rpm for 2.5 min to remove particulate and biological matter and then diluted 1:100 for final analysis. The pump flow rate was 1 mL/min; injection volume of the samples was 25 μL and a column temperature of 30 °C was used. A hydroxide eluent gradient method was established for separation of anions. The KOH gradient started with initial concentration of 1.7 mM for 8 min and then incremental increases from 1.7 to 14 mM from 8 to 11.25 min, then incremental increases from 14 to 28 mM from 11.25 to 11.3 min, then held at 28 mM until 19 min, then dropped to 1.7 mM at 19.1 min and held up to 22 min. Calibration standards with known concentrations of acetate, propionate, formate, lactate and sulfate were prepared as well and used to quantify the different samples.

### Weight loss corrosion rate measurement

A passive Clarke solution was used to remove the residual corrosion products or scale on the coupon surface for general corrosion rate measurement. Stannous chloride (1.25 g), antimony trioxide (0.5 g) and hydrochloric acid (25 g, or ~ 21 mL of 36–38% conc. hydrochloric acid) were combined in a 100 mL beaker and mixed well. Test coupons were placed into the solution and swirled for 20 s. Nonmetallic tweezers were used to remove the coupon from the solution, following which, it was rinsed with water, followed by acetone and then blow dried with nitrogen. Each specimen was weighed three times. The same passive Clarke solution was used for up to 2 h, after which, a new solution was made. Weight was recorded and the cleaning and weighing process was continued in the same manner until no more weight loss could be detected. Average weight from the last measurement was recorded as final weight of the test coupon. General weight loss corrosion rate of each coupon was determined by measuring the difference between pre and post-test coupon weights, and the post-test weight measurement was performed using a procedure based on the ASTM G1-03 standard. Triplicate weights of descaled metal coupons were recorded to calculate corrosion rates (CR) in mm/y, using the formula: *CR* = *kw/* (*Atρ*) where *k* is a constant (*k* = 8.76 × 10^4^), *w* is the weight loss (initial − final weigh) in grams, *A* is the surface area of the metal coupons in cm^2^, *t* is the incubation time in hours, and *ρ* is the density of the carbon steel in g/cm^3^ (7.85 g/cm^3^ for X-52 carbon steel).

### Extracellular protein precipitation with DOC-TCA protocol

Planktonic bacterial cultures were filtered through 0.2 µM cellulose acetate filters to obtain extracellular samples from the test cultures. 150 µL of 0.15% sodium deoxycholate (DOC) solution was added to 1.5 mL of the filtered test solution. The mixture was vortexed and incubated at room temperature for ≥ 15 min. 160 µL of 100% trichloroacetic acid (TCA) was added and a fine precipitate was formed in the mixture, the final concentration of TCA in the mixture was ~ 10%. The mixture was vortexed and incubated at −20 ℃ for ≥ 15 min or overnight. The mixture was then centrifuged at maximum speed for 10 min at 4 ℃ and immediately and carefully the supernatant was decanted and the pellet was retained. 1 mL of 100% ice cold acetone was added and mixed well by vortexing to wash out the residual TCA. The mixture was centrifuged at maximum speed for 10 min at 4 ℃. The supernatant was decanted and the tube was dried by inverting on tissue paper. The pellet was directly sent to Princeton University on dry ice for LC–MS/MS analysis.

### Collection of biofilm cells from coupon

Test coupons made of carbon steel were collected in sterile 15 mL falcon tubes containing sterile 1X phosphate buffered saline (PBS) solution (5 mL, pH 7.4). The mixture was sonicated in a Branson 2510 ultrasonic bath containing ice-water for 40 min to sonicate biofilm cells off the test coupon into the sterile 1X PBS. The cell suspension was then centrifuged in a Sorvall ST40R centrifuge at 3500 rpm, 4 °C for 40 min to pellet the biofilm cells. The supernatant was discarded and the cells were frozen at − 80 °C.

### Corrosion product characterization

After biofilm removal, the coupons with remaining biomass and corrosion products were further vortexed at maximum speed for an additional 5 min in acetone. Subsequently, the acetone phase was filtered using Anodisc filter membrane (47 mm in diameter with pore size of 0.2 µm) and the corrosion products were collected and analyzed by a Rigaku MiniFlex II X-ray diffraction (XRD) system for phase identification. The coupons, now free of biofilm and loose corrosion products, were examined with a LEO Gemini 1530 scanning electron microscope (SEM), along with energy dispersive X-ray spectroscopy (EDS) for surface residual corrosion product analysis. Coupons with corrosion product or scale left on the surface after biofilm removal were also analyzed by XRD to identify the crystal structure of the corrosion products. After the corrosion product analysis, the scale on the coupon surface was removed from each coupon using a passive Clarke solution and the weight of the descaled coupon was measured for general weight loss corrosion rate, as described above.

### Biofilm imaging with SEM

Scanning electron microscopy was used to characterize *D. ferrophilus* IS5 biofilm interactions with carbon steel surfaces. *D. ferrophilus* IS5 cultures were grown anaerobically in laboratory serum bottles as biofilms attached to carbon steel coupons suspended within anaerobic MIC media. After eight weeks, the carbon steel coupons, with attached IS5 biofilm were retrieved from culture bottles, rinsed briefly in 0.1 M cacodylate buffer (pH 7.4 adjusted with H_2_SO_4_) and prepared for imaging on a Zeiss Crossbeam 540 SEM (Zeiss, Germany). Biofilms attached to coupons were fixed using chemical fixation and chemical critical point drying techniques. First, the coupons were placed in a solution composed of 25 mM lysine, 4% paraformaldehyde (PFA), and 2.5% glutaraldehyde (GA) in 0.1 M Na-cacodylate (CA) buffer (pH 7.4) and left to soak for 2 h, undisturbed. Samples were then transferred to a solution of 2.5% GA in 0.1 M CA buffer for 24 h. Stable, cross-linked samples were rinsed with 0.1 M CA prior to sequential dehydration protocol.

Sequential dehydration was conducted using incrementally graded concentrations of ethanol in de-ionized water, starting with 40%, 50%, 75%, 85%, 95%, and three times at 100%. At each concentration, the sample was left for 20 min. Chemical critical point drying was used to complete dehydration by first soaking samples in a 1:1 solution of hexamethyldisilazane: ethanol for 20 min. Finally, dehydration was completed by soaking the samples 2 times (20 min/time) in 100% hexamethyldisilazane (HMDS) and allowing the liquid solution to evaporate completely. Once completely dry, the carbon steel coupon with fixed biofilm was securely attached to a 12 mm aluminium stub and sputter coated with gold.

### LC–MS/MS mass spectrometry analysis

An Orbitrap Fusion Lumos Tribrid mass spectrometer interfaced with Easy nLC-1200 ultra-high-pressure liquid chromatography system was used for LC–MS/MS process. The Orbitrap Fusion Lumos Tribrid mass spectrometer was set with a scan range of 375–1500 (m/z) at resolution of 120,000. Nano spray ionization in positive mode with capillary voltage of 1800 V and ion transfer tube temperature of 275 °C was used. Data-dependent acquisition method (DDA) was performed. The parent MS1 data were collected as profile type. AGC gain was set at 4.0e5 with 50 ms maximum injection time. Multiple charge states from 2 to 7 are included for MS/MS fragmentation. HCD activation with 1.2 m/z isolation window and 35% HCD collision energy were also used. MS/MS daughter ions were detected in linear ion trap with 1.0e4 AGC target. Maximum injection time was 45 ms and data was collected in centroid mode. Rapid ion trap scan rate was used. Dynamic exclusion duration was set at 60 s and mass tolerance was set at 10 ppm. The cycle time between Master-scans is 3 s. The total run time was 130 min.

Easy nLC-1200 UHPLC flow was set at 300 nL/min. Maximum pressure was set at 720 bar. Buffer A is 0.1% formic acid in water and buffer B is 0.1% formic acid in 80% acetonitrile/20% water. The 75 µm id × 50 cm long fused silica Nano column was packed with R119-AQ-003 C18 resin. Column temperature was 45 °C. Gradient from 2% B to 27% B was run in 123 min for peptide separation followed by 10 min 95% B wash. Auto-sampler flush volume is 100 µL. To minimize carry over from previous sample, two one-hour blank runs were included between samples. The peptide amount of trypsin-digested sample was measured by Nano-Drop UV spectrometer. Appropriate volume of diluent (0.1% formic acid in water) was added to get a final concentration of 180 ng/µL. Injection volume was set at 2 µL.

### LC–MS/MS data analysis for protein identification

Proteome Discoverer software (Version 2.2, Thermo Fisher Scientific, San Jose, CA, https://proteomesoftware.com/) was used for data analysis. Two separate databases of *D. ferrophilus* IS5 were downloaded. The first database UP000269883 (TrEMBL/unreviewed) was downloaded from UniProt (www.uniprot.org). The second set of databases, AP017378.1 and AP017379.1 were downloaded from Genbank (https://www.ncbi.nlm.nih.gov/nuccore) and combined into one database. Raw LC–MS/MS results were searched against *D. ferrophilus* IS5 databases from Uniprot and Genbank separately using Sequest HT search engine (Thermo Fisher Scientific, San Jose, CA). Parent (MS1) ion mass tolerance of 10 ppm and fragment (MS2) ion mass tolerance of 0.40 Da was used. Trypsin enzyme was chosen. Carbamidomethylation of cysteine was specified as fixed modification. Oxidation of methionine, deamidation of asparagine and glutamine were set as dynamic modifications. Pyro glutamate formation from glutamic acid and glutamine was specified as dynamic modification at peptide N-terminus. Acetylation was set as dynamic modification at protein N-terminus. Scaffold version 4.8.8 (Proteome Software Inc., Portland, OR) was used to validate MS2 based peptide and protein identifications. Peptide identifications were accepted if they could be established at greater than 95.0% probability. Protein identifications were accepted if they could be established at greater than 99.0% probability and contained at least 2 identified peptides. Proteins were identified with the following parameters in Scaffold Viewer: protein threshold of 99%, number of minimal peptides at 2, and peptide threshold of 95%. Normalized spectral abundance factor (NSAF) based spectral count was adopted for quantification of the complex proteomics^19^. Statistical significance thresholds of a t-test *p*-value of 0.05 was applied for the quantitative analysis.

## Supplementary Information

Supplementary Figures.

Supplementary Information 1.

Supplementary Information 2.

Supplementary Information 3.

## Data Availability

All data generated or analyzed during this study are included in this published article (and its Supplementary Information files). The mass spectrometry proteomics data have been deposited to the ProteomeXchange Consortium via the PRIDE^20^ partner repository with the dataset identifier PXD026513 and 10.6019/PXD026513.
