# Supplementary material for: Proteomic study of Desulfovibrio ferrophilus IS5 reveals overexpressed extracellular multi-heme cytochrome associated with severe microbiologically influenced corrosion
Source: Sci Rep. 2021 Jul 29;11:15458. doi: 10.1038/s41598-021-95060-0 (PMC8322314; doi:10.1038/s41598-021-95060-0)
Supplement: Supplementary file 1 — Supplementary Figures. [file 41598_2021_95060_MOESM1_ESM.docx]

**Proteomic study of *Desulfovibrio ferrophilus* IS5 reveals overexpressed extracellular multi-heme cytochrome associated with severe microbiologically influenced corrosion**

Mohor Chatterjee^1^, Yu Fan^2^, Fang Cao^1^, Aaron A. Jones^1^, Giovanni Pilloni^1^ & Xiaozhou Zhang^1*^

^1^Corporate Strategic Research, ExxonMobil Research and Engineering Company, 1545 Route 22 East, Annandale, New Jersey 08801, United States. ^2^ExxonMobil Technical Computing Company, 1545 Route 22 East, Annandale, New Jersey 08801, United States.

^*^email: xiaozhou.zhang@exxonmobil.com


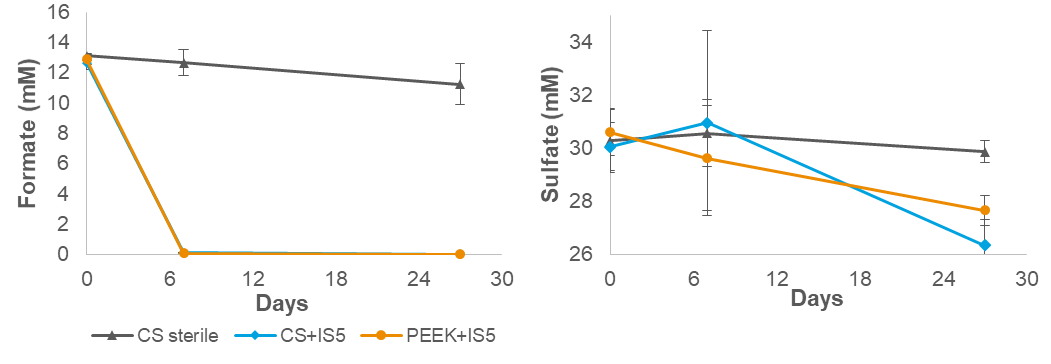


**Supplementary Figure 1.** Metabolic profiles of *D. ferrophilus* IS5 and corresponding sterile control. The error bars represent the calculated standard deviations of the measurements of three biological replicates.


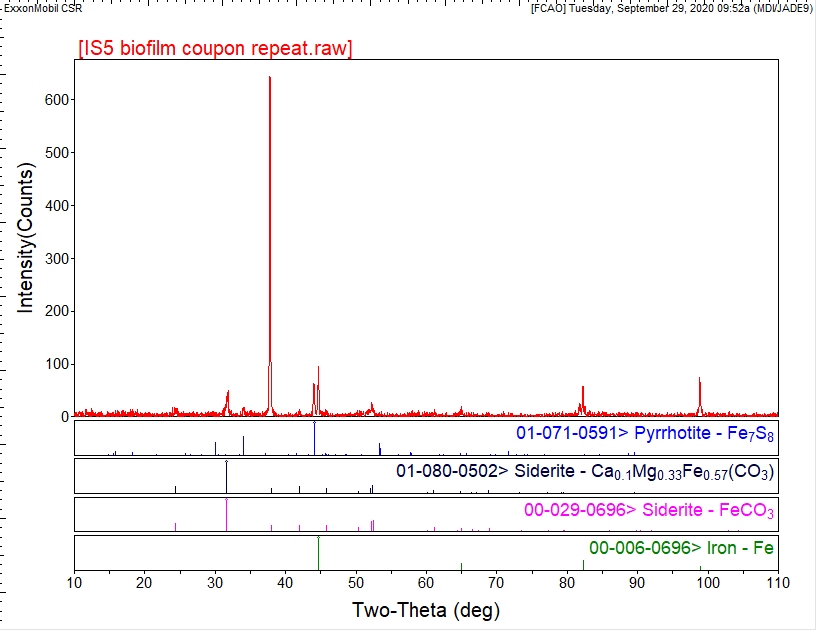


**Supplementary Figure 2.** XRD analysis result of the residual corrosion products on steel coupon surface after biofilm removal, suggesting the presence of siderite and pyrrhotite.


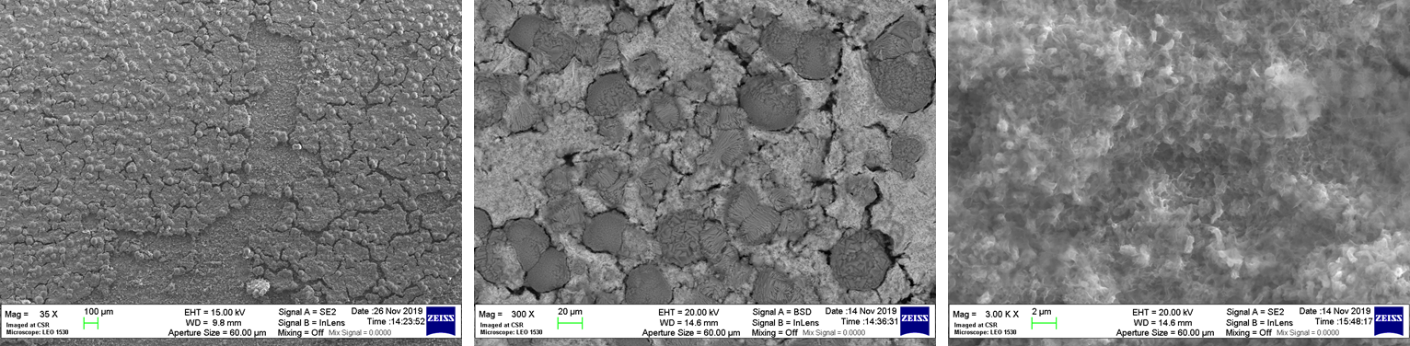


**Supplementary Figure 3**. SEM images of the residual corrosion products on the CS coupon surface after biofilm removal with increasing image magnification from left to right.


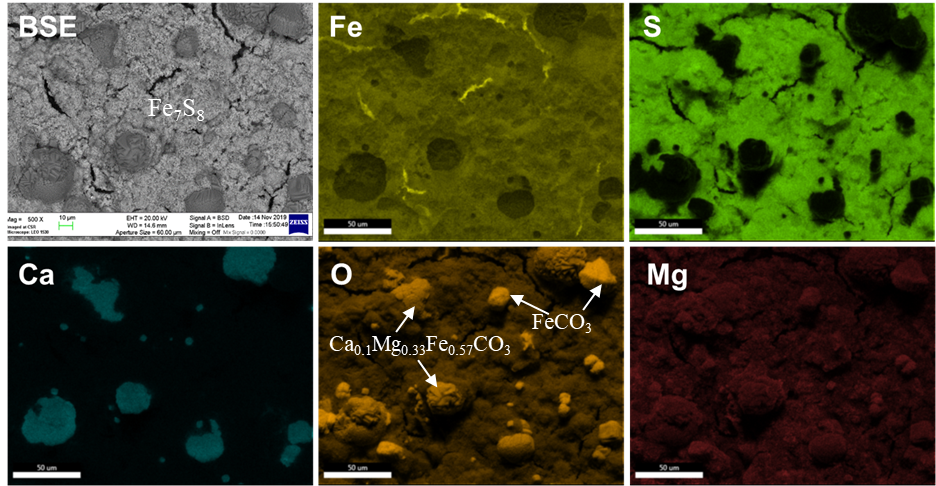


**Supplementary Figure 4**. Backscattered electron (BSE) image of the corrosion products on the coupon surface and corresponding EDS elemental maps.


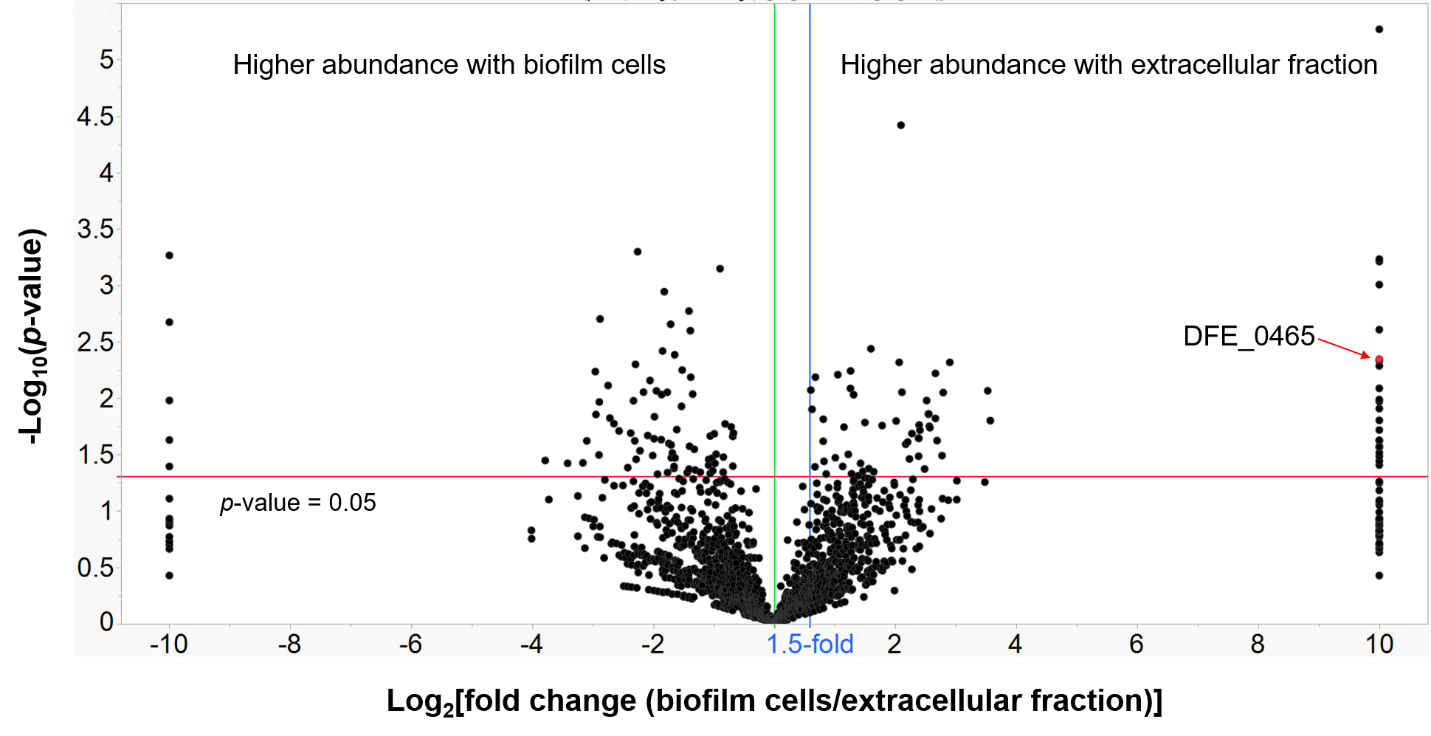


**Supplementary Figure 5**. Volcano plot representing the results of the proteome analysis of proteins (extracellular fraction versus biofilm cells) with *D. ferrophilus* IS5 cultured with either CS coupon. Highlighted (red) point represents the cytochrome DFE_0465 showed higher abundance in the extracellular environment when the culture was incubated with the CS coupon and showed severe EMIC, with statistical significance. Red lines represent the applied significance thresholds of a t-test *p*-value = 0.05. Blue lines represent the applied thresholds of an absolute fold change ≥ 1.5.
